# Role of Macrophages in the Pathogenesis of Atopic Dermatitis

**DOI:** 10.1155/2013/942375

**Published:** 2013-03-04

**Authors:** Sadaf Kasraie, Thomas Werfel

**Affiliations:** ^1^Global Preclinical Drug Discovery, Department of Molecular Pharmacology, Grünenthal GmbH, Zieglerstrße 6, 52078 Aachen, Germany; ^2^Division of Immunodermatology and Allergy Research, Department of Dermatology and Allergy, Hannover Medical School, 30625 Hannover, Germany

## Abstract

Atopic dermatitis (AD) is one of the most common and most intensively studied chronic inflammatory skin diseases. Several cofactors, such as an impaired skin barrier function, modifications of the immune system, and a complex genetic background, direct the course of AD. Within this complex network, macrophages play a pivotal role in enhanced susceptibility to cutaneous infections and act as central connecting components in the pathogenesis of AD on the cellular level. In AD, macrophages are known to accumulate in acutely and chronically inflamed skin. During the early and short inflammatory phase, macrophages exert proinflammatory functions like antigen-presenting phagocytosis and the production of inflammatory cytokines and growth factors that facilitate the resolution of inflammation. However, persistence of pro-inflammatory activity and altered function of macrophages result in the development of chronic inflammatory diseases such as AD. The exact mechanism of macrophages activation in these processes is not yet completely understood. Further studies should be performed to clarify the dysregulated mechanism of macrophages activation in AD, and this would allow us to target these cells with versatile functions for therapeutic purpose and improve and control the disease. In this paper, we highlight the new findings on dysregulated function of macrophages and the importance of these cells in the pathogenesis of AD in general and the contribution of these cells in enhanced susceptibility against microbial infections in particular.

## 1. Introduction

Besides providing a structural barrier, the skin contains several immune cells that can be activated by invading pathogens or skin damage. One of the most important immune cells involved in inflammation and wound healing is the macrophage, which exhibits different immunological functions in the skin, including phagocytosis and antigen presentation. Furthermore, macrophages produce many cytokines and chemokines that stimulate new capillary growth, collagen synthesis, and fibrosis [1]. This immune cell is thought to orchestrate the resolution of inflammation and the wound healing process throughout the different phases such as haemostasis, inflammation, proliferation, angiogenesis, and reepithelialisation as well as remodeling [1–3].

Researchers have long known that macrophages residing in or migrating to different tissues or sites of infection and damage have distinct appearances and cell surface phenotypes; for example, Kupffer cells (liver resident macrophages) appear microscopically different than splenic red pulp macrophages. Until recently, phenotyping macrophages and other related mononuclear phagocytes, including the many dendritic cells (DCs) subtypes, with cell surface markers such as CD11b, CD68, macrophage antigen-2, and F4/80, has been the mainstay of macrophage characterization. However, the last decade has provided new ways of phenotyping macrophages based on their gene-expression profile in response to specific stimuli. By far, the most often-used terms in gene-expression-based macrophage phenotyping are classically activated macrophages (CAMs) (also called M1) and alternatively activated macrophages (AAMs) (M2), which are thought to have characteristic gene-expression profiles defined by markers linked to the stimulation conditions used to generate the subtype—toll-like receptor (TLR) stimulation, bacterial infection, and interferon-(IFN-)*γ* stimulation for CAMs and IL-4/IL-13 for AAMs. It is not surprising that given tendencies of immunologists for cell categorization, CAMs and AAMs have been atomized into smaller tranches such as M1a and M2a and M2b. A major question, therefore, concerns the function of the different macrophage types in different homeostatic, infection, and tissue-repair scenarios. Surprisingly, little is known about the functions of individual AAM-associated genes in comparison with CAM-associated macrophage-inflammatory and tissue-remodeling products. However, the gap in knowledge concerning AAM effector functions is closing rapidly with recent publications investigating the effects of deletion of two AAM-associated effector genes, *Arg1* and Retnla. Furthermore, correlations between mouse and human tissue macrophages and their representative subtypes are lacking and are a major barrier to understanding human immunity [4].

Macrophages play key roles in inflammation [5]. During the onset of the inflammatory process, these phagocytic cells become activated and have destructive effects. Macrophage activation, which involves the induction of more than 400 genes, results in an increased capacity to eliminate bacteria and to regulate many other cells through the release of cytokines and chemokines. However, excessive activation has damaging effects, such as septic shock, which can lead to multiple organ dysfunction syndrome and death. In other situations, persistence of proinflammatory activity results in the development of chronic inflammation including chronic inflammatory skin diseases such as psoriasis and atopic dermatitis (AD) [5].

AD is one of the most frequent chronic inflammatory skin diseases with an increasing prevalence affecting 10%–20% of children and 1%–3% of adults in industrial countries [6, 7]. It has a significant impact on the quality of life of patients and their families, and the economic impact is estimated to be billions of dollars [8]. 

Patients with AD have frequent bacterial and viral skin infections. The most predominant bacteria on AD skin are *Staphylococcus aureus*, constituting 90% of the bacterial microflora on lesional skin and importantly colonizing normal-appearing skin [9]. Many studies have shown that the extent of *S. aureus* colonization positively correlates with the disease activity of AD [10]. 

Several cofactors, such as an impaired skin barrier function, modifications of the immune system, and a complex genetic background, direct the course of AD [11–13].

Activation of T lymphocytes, DCs, macrophages, keratinocytes, mast cells, and eosinophils is characteristic of AD skin inflammatory responses.

Clinically unaffected skin in AD is not normal. It is frequently dry and has a greater irritant skin response than normal healthy skin. Microscopic studies reveal a sparse perivascular T-cell infiltrate in unaffected AD skin that is not seen in normal healthy skin. Acute AD skin lesions present to the physician as intensely pruritic, erythematous papules associated with excoriation and serous exudation. There is a marked infiltration of CD4^+^ activated memory T cells in acute AD. Antigen-presenting cells (e.g., Langerhans cells (LCs), inflammatory dendritic epidermal cells (IDECs), and macrophages) in lesional and, to a lesser extent, in nonlesional skin bear IgE molecules. Mast cell degranulation can be observed.

Chronic AD skin lesions have undergone tissue remodeling caused by chronic inflammation. These skin lesions are associated with thickened plaques with increased skin markings (lichenification), increased collagen deposition in the dermis, and dry fibrotic papules.

Macrophages dominate the dermal mononuclear cell infiltrate. Eosinophils also contribute to the inflammatory response, and T cells remain present, although in smaller numbers than seen in acute AD [14].

Within this complex network, antigen-presenting cells such as dendritic cells (DCs) and macrophages play a pivotal role as central connecting components on the cellular level.

Monocytes are important previous cells of macrophages that are involved in skin inflammation of AD [15]. Monocytes invade the dermis and differentiate into macrophages, which can also act as antigen-presenting cells (APCs) [16]. 

In AD, macrophages are known to accumulate in acutely and chronically inflamed skin [17]. In this paper, we highlight the new findings on dysregulated function of macrophages and the importance of these cells in the pathogenesis of AD in general and the contribution of these cells in enhanced susceptibility against microbial infections in particular.

## 2. Tissue-Specific Macrophage during Cutaneous Inflammation in AD 

Mononuclear phagocytes include tissue-resident cells, such as macrophages and DCs as well as blood monocytes and myeloid progenitors. These progenitors travel through the blood and lymphatic circulation to seed both lymphoid and nonlymphoid tissues, where they develop further, acquiring specific effector functions. DCs are uniquely specialised to detect perturbations originating from both outside and inside the organism. DCs possess the capacity to respond to infectious or noninfectious stress signals, and, following stimulation, they first initiate and then regulate adaptive immunity. DCs function is highly plastic; they can adapt their functional characteristics appropriately, when homing to tissue microenvironments as varied as the skin, the lung, or the gut mucosa. Similarly, when macrophages seed different tissues, they must also adapt and respond to the specific microenvironment. Macrophages and DCs are derived from myeloid bone marrow progenitors and reach the tissues via the blood, yet occupy distinct functional niches; so, it is highly pertinent to determine their precise lineage and progenitors. Once identified, it should be possible to answer long-standing questions concerning when and where in the body specific DC or macrophages commitment occurs and so better understand their differing immune properties *in vivo* and perhaps how they might be better manipulated therapeutically. 

**Figure 1:**
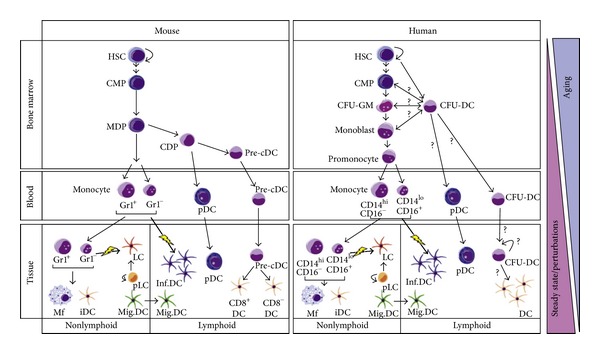
Mononuclear phagocyte differentiation in mouse and human. Hematopoiesis in mouse and human begins from the hematopoietic stem cells (HSCs). The HSCs are self-renewing with clonogenic and multipotent features, giving rise to all blood and tissue-resident immune cells. A very small number of HSCs generate immune cells *de novo* through a multistep differentiation process passing through multilineage progenitors first and to committed progenitors later. All of this process is strictly regulated according to physiological requirements, be it in the steady state or in response to external perturbations, such as infection. Aging also profoundly affects the function of the immune system. The phenomenon is attributable mainly to changes in the HSC compartment that probably gradually reduces its capacity for self-renewal, leading to a progressive reduction in the numbers of immune cells. Myeloid differentiation starts from a common myeloid progenitor (CMP) in the bone marrow. In the mouse, the destiny of CMP is better characterized compared to that of the human counterpart. The CMP generates macrophage-(Mf-) dendritic cell (DC) precursors (MDPs), which are considered the direct progenitor of Gr1^+^ and Gr1^−^ monocytes in the blood. Besides monocytes, the MDP differentiates into the common DC precursor (CDP), which in turn generates plasmacytoid DC (pDC) and progenitors for conventional DC (pre-cDC). pDC and myeloid DC diverge at the CDP stage. Pre-cDCs migrate out of the BM through the blood circulation into secondary lymphoid tissues (spleen and lymph nodes), where they replenish both CD8^+^ and CD8^−^ DC in the tissues. In humans, CMPs differentiate to granulocyte-macrophage (CFU-GM) precursor, which give rise to monocytes (CD14^hi^CD16^−^ and CD14^lo^CD16^+^) through subsequent differentiation steps, monoblast first, followed by promonocyte. A DC precursor (CFU-DC) derived from CD34^+^ HSC with unique differentiation potential towards DC has been identified in bone marrow of the mouse, suggesting that a DC progenitor might exist in humans. Blood monocytes (Gr1^+^ and Gr1^−^ in mouse, CD14^hi^CD16^−^ and CD14^lo^CD16^+^ in humans) migrate to nonlymphoid tissues and generate macrophages (Mfs) and interstitial DC (iDC). In the presence of environmental perturbation *in vivo* or of cytokines *in vitro*, Gr1^+^ or Gr1^−^ monocytes in mice and CD16^+^ or CD16^−^ monocytes in humans differentiate into Langerhans cells (LCs) in the epidermis, as well as inflammatory DC (Inf.DC). LCs are generated by a dermal long-term precursor (pLC) in the steady state. Finally, migratory DC (Mig.DC) moves between nonlymphoid and lymphoid compartments.

Analysis of the origins of mononuclear phagocytes and their pathways of differentiation have been hampered for decades by a lack of molecular markers with defined specificity for particular precursors or subpopulations. To date, much of our understanding of human DC is based on *in vitro* generated cells; however, it is still unknown to what extent they faithfully reproduce the phenotype and function of tissue DC. There is an urgent need to identify and characterise DC progenitors from human blood and to use these “untouched” cells to better understand specific DC functional capabilities. It is possible that certain blood monocyte subpopulations, such as CD14^+^CD16^+^ monocytes, might retain some functional characteristics of DC; for example, they can exhibit enormous plasticity and heterogeneity and may have a role in a range of human diseases (Figure 1) [18].

Macrophages and DCs may play a role in chronicity of AD [11]. However, so far, only limited data are documented on the distribution of macrophages in the skin during cutaneous inflammation. 

Kiekens et al. [17] characterized monocytes-derived cells in affected lesional AD skin, compared with nonaffected AD skin and healthy skin. They showed that there was an increase in macrophage numbers in acutely and chronically inflamed AD skin, whereas absolute DC numbers were unchanged, compared with nonlesional AD skin. 

The macrophage markers RFD7 (mature tissue phagocyte marker) and CD68 show similar expression patterns during acute and chronic cutaneous inflammation. The total number of RFD7^+^ macrophages was lower than the number of CD68^+^ macrophages [17].

Healthy human skin macrophages are known to express CD36, and functionally CD36 is linked to phagocytosis of apoptotic cells [19, 20]. 

In acutely and chronically inflamed AD skin, Kiekens et al. [17] found increased expression of CD36 by macrophages. In inflamed tissue, many immune cells go into apoptosis after fulfilling their effector function and need to be removed efficiently. Increased expression of CD36 by macrophages may be linked to the removal of apoptotic cells [17].

Human monocyte-derived DCs express mannose receptors (MRs), as was shown by *in vitro* studies, and these cells use the MR for efficient antigen uptake [21]. In peripheral tissues such as the skin, antigen uptake is an important feature of resident macrophages and immature DCs. Both macrophages and DCs express MR in cutaneous inflammation; in nonlesional skin, their number is significantly increased compared with healthy skin. MRs are expressed mainly by macrophages in inflamed AD skin [17]. 

This can be explained by the fact that macrophages and not DC numbers increase in inflamed AD skin.

Furthermore, phenotypically heterogeneous and overlapping macrophage and DC populations are present in inflamed AD skin. The classic macrophage marker CD68 and prototypic DC marker CD1a could bind to the same cell subpopulation in the dermis of inflamed AD skin [17]. Kiekens et al. [17] demonstrated that, within tissue-specific macrophage populations, further subpopulations are present and that monocyte-derived cells may express markers for both DCs and macrophages. Their results point to the existence of a heterogeneous pool of macrophage/dendritic cell-like cells, from which subpopulations of dermal macrophages and DCs arise [17]. 

A recent study by Sugaya et al. [22] indicates that the numbers of CD163^+^ cells (alternatively activated macrophages marker) in lesional skin of AD were significantly larger than in normal skin. Interestingly, the number and distribution of CD163^+^ cells were quite similar to those of CD68^+^ cells which were consistent with a previous report [22].

Since in AD research most emphasis has been put on the regulatory role of T cells, little attention has been paid to the monocyte-derived macrophages and their potential role; no conclusive data are available on the distribution and clear phenotype of these cells in the skin of AD patients.

Therefore, further studies should be conducted in order to address the exact function of macrophages during different phases of the skin inflammation.

## 3. Phagocytosis 

Individuals with AD frequently present recurrent infections from pyogenic bacteria or from intracellular microorganisms. The mononuclear and polymorphonuclear neutrophilic phagocytes participate in the innate defense, acting quickly against different agents. These cells initially present chemotactic activity, migrating towards the chemotactic factors and then to the area where the immune response takes place. Following this, phagocytosis occurs, which consists in the ingestion and digestion of the pathogenic organisms, with subsequent elimination of their inactivated products [23]. 

The high frequency of infections in individuals with AD suggests immune disorders, possibly involving the alterations of neutrophilic and mononuclear phagocytes. However, these alterations have not been fully understood in monocyte-derived macrophages [23, 24]. 

Forte et al. [25] observed a deficiency in the activity of mononuclear phagocytes in five patients with AD [25]. In another study, they evaluated phagocytes in 19 patients with AD and demonstrated that there was a reduction in the phagocytic activity by mononuclear phagocytes in patients with AD in all age groups studied. In the case of neutrophils, the same deficiency was observed only in patients with AD over 12 years of age [23].

Their data demonstrated a reduction in chemotactic response and phagocytic activity by neutrophilic and/or mononuclear phagocytes in the majority of patients with moderate to severe AD. Their results were coherent with the clinical data concerning the higher incidence of infections by pyogenic bacteria and fungi in patients with AD, which are microorganisms that require defense by the phagocytes [23]. 

The recurrent infections by pyogenic bacteria or by intracellular organisms that occur in AD suggest that phagocytic activity disorders occur with greater frequency. 

## 4. Pattern Recognition Receptors (PRRs)

The major players in the detection of invading pathogens are the recently identified TLRs. The success of TLRs to function as major sensors of invading pathogens is their ability to identify a range of conserved microbial motifs termed “pathogen-associated molecular patterns” (PAMPs). Innate recognition of PAMPs by TLRs can initiate a cascade of signaling pathways that eventually culminate in the induction of a wide range of immune and inflammatory genes. The most important products of these genes include chemokines and adhesion molecules which result in the recruitment of circulating monocytes from the bloodstream and the production of inflammatory cytokines such as tumour necrosis factor (TNF), interleukin-1 (IL-1), and IFN which mount an inflammatory immune response. As well as their initiation of the innate immune response, there is increasing evidence to suggest that TLRs can also play a role in other macrophage functions such as phagocytosis, antigen processing, and presentation and initiation of the adaptive immune response [26].

Evidence has shown that most of the ten TLRs are expressed on macrophages. In an early study where the mRNA expression of TLRs 1–5 was analysed in a fresh human leukocyte population containing monocytes, T lymphocytes, natural killer (NK) cells, DC, and polymorphonuclear (PMN) cells, TLR-1 was found to be ubiquitously expressed, whereas TLR-2, TLR-4, and TLR-5 were found on monocytes, DCs, and PMNs, and the expression of TLR3 appeared to be exclusively expressed on DCs [27, 28]. Although macrophages were not analysed in this study, it is important to note that the expression of TLRs on monocytes can induce their activation so that they differentiate into either macrophages or DCs [29]. Further analysis has revealed that TLR-6, TLR-7, and TLR-8 are also expressed on freshly isolated human monocytes, whereas TLR-9 and TLR-10 have been shown to be expressed on certain subsets of human DCs [29, 30]. To add to the complexity, TLR expression appears to differ between mouse and human. For example, human TLR-3 appears to be exclusively expressed on DCs, whereas it is expressed and strongly induced in macrophages from mice. TLR-4 is expressed strongly on monocytes and macrophages in both species; however, TLR-4 mRNA expression increases upon LPS stimulation in human macrophages, whereas TLR-4 mRNA is downregulated in response to LPS in murine macrophages [31]. In addition, TLR-9 appears to be almost exclusively expressed on plasmacytoid DCs in both humans and mice; however, in response to LPS, TLR-9 expression can be upregulated in murine macrophages [29, 32]. Mice fail to express TLR-10; however, they express additional TLRs such as TLR-11, TLR-12, and TLR-13 which are absent in humans [33].

The mechanisms that promote the enhanced susceptibility to cutaneous infections in AD are complex interactions among several factors. These contributing factors include skin barrier dysfunction, reduced skin lipid content, and abnormalities of the innate immune response. Some of the innate immune defects observed in AD are primary defects such as epithelial barrier defects and defects in signaling or expression of innate receptors. Others may be secondary to the effects of the adaptive immune response. For example, deficiencies in antimicrobial peptides (AMPs) may be due to the overexpression of Th2 cytokines such as interleukin-(IL-) 4 and IL-13 [7, 34].

The innate immune system protects the host from pathogens and initiates the repair process following injury or trauma. It senses microbes through a group of germline encoded proteins, named pattern-recognition receptors (PRRs). 

Host recognition of bacterial pathogens including *S. aureus* is mediated in part by PRRs, including membrane-bound toll-like receptors (TLRs) and intracellular nucleotide-binding oligomerization domain receptors (NLRs).

TLRs act as PRRs comprising a family of at least 10 receptors in humans with distinct recognition profiles [35]. In this context, TLR-2 has emerged as a principle receptor in combating Gram-positive bacteria, especially *S. aureus* [36, 37]. Of the key cells which express TLR-2 are monocytes and macrophages, and they contribute to eliminate pathogens.

TLR-2 forms heterodimers with TLR-1 and TLR-6 to interact with a rather broad spectrum of ligands. Studies using knockout mice identified TLR-1 as the coreceptor required for the recognition of triacylated lipoproteins and lipopeptides such as Pam3Cys [36]. Diacylated components such as lipoteichoic acid (LTA), which is a component of the cell wall of *S. aureus*, interact with TLR-2/TLR-6 heterodimers [36, 37]. Peptidoglycan is a major constituent of the cell wall of Gram-positive bacteria, which induces signal transduction via TLR-2, nucleotide-binding oligomerization domain (NOD) 1 (card4), and NOD2 (card15) receptors, respectively.

NOD molecules, including NOD1 and NOD2, are a family of intracellular pattern recognition proteins involved in bacterial detection [38, 39]. In this context, children with impetiginized AD were found to have increased levels of the TLR-2 ligand LTA in lesional skin that correlated with lesional Eczema Area and Severity Index scores and *S. aureus* colony-forming units. Importantly, the amounts of LTA detected in lesional skin were sufficient to exert biological effects on various cell types *in vitro* [40]. 

There is emerging evidence that supports a general impairment of TLR-2 expression and TLR-2-mediated proinflammatory cytokines in monocytes and macrophages from AD patients [41, 42].

We could show that macrophages from patients with AD expressed significantly less TLR-2 compared with healthy controls, whereas the expression patterns of TLR-1 and TLR-6 were not altered. Macrophages had a reduced capacity to produce proinflammatory cytokines such as IL-6, IL-8, and IL-1*β* after stimulation with TLR-2 ligands, which might contribute to the enhanced susceptibility to skin infections with *S. aureus* in AD [42]. Interestingly, weak TLR-2 and TLR-4 signals in the context of allergen exposure in the skin and lung, respectively, had previously been shown to promote a Th2-biased immune response [43]. Therefore, weak TLR-2 responses may not only render AD patients incapable of eradicating *S. aureus* colonizing their skin, but may also promote a Th2 response.

Genetic TLR-2 polymorphisms have been shown to affect the severity of AD. A high frequency (12%) of adult AD patients was found to carry the TLR-2 R753Q single-nucleotide polymorphism (SNP). These patients suffered from a more severe phenotype compared with AD patients without this mutation [44]. These data suggest that the TLR-2 polymorphism R753Q increases the susceptibility to infections and chronic colonization with various pathogens, including *S. aureus.*

In addition, we could show functional differences in TLR-2 responsiveness of monocytes from AD patients with the TLR-2 R753Q mutation compared with wild-type AD patients [45]. 

More recently, Nod2, an NLR protein which senses muramyl dipeptide produced during the synthesis and/or degradation of peptidoglycan, has been implicated in the host response to *S. aureus* [46]. While TLR2 and Nod2 induce immune responses via the activation of the transcription factor NF-*κ*B and MAP kinases [47], another group of NLRs that include Nlrp3 and Nlrc4 are critical for the activation of caspase-1 and IL-1*β* secretion in response to bacterial and endogenous stimuli in macrophages [48]. 

Previous studies have shown that IL-1*β* signaling and the adaptor protein Asc play a critical role in the clearance of *S. aureus* infection in the skin through monocytes and macrophages [49]. 

Muñoz-Planillo et al. [49] showed that *S. aureus* hemolysins including *α*-toxin circumvent the requirement of ATP and the P2X7 receptor to induce caspase-1 activation in macrophages via Nlrp3 inflammasome. 

We recently showed that staphylococcal *α*-toxin contributes to the Th1 polarization by induction of CXCL10 in macrophages. However, macrophages from patients AD show a reduced CXCL10 expression in response to staphylococcal *α*-toxin [50]. Our data support the hypothesis that the contribution of macrophages in the pathogenesis of AD is linked to the presence of distinct alterations in their capacity to respond to the staphylococcal exotoxin *α*-toxin and that these abnormalities can modulate the amplification and persistence of chronic skin inflammation [50]. 

The mechanism of macrophages activation by staphylococcal *α*-toxin through inflammasome in monocytes and macrophages from patients with AD is not investigated yet.

Inflammasome-dependent mechanisms which may be altered in patients with resistant AD may contribute to the chronification of the disease and the susceptibility of patients with AD to cutaneous microbial colonization and infections.

Taken together, these data partially explain how macrophages contribute to skin colonization and infection with *S. aureus* and play a crucial role in chronic skin inflammation in AD.

## 5. The Cytokine and Chemokine Network

Monocytes and macrophages are source of many cytokines and chemokines which play a fundamental role in pathogenesis of many chronic inflammatory diseases such as AD (see Table 1) [51–53]. 

Diversity and plasticity are hallmarks of cells of the monocyte-macrophage lineage. In response to IFNs, TLR engagement, or IL-4/IL-13 signaling, macrophages undergo M1 (classical) or M2 (alternative) activation, which represent extremes of a continuum in a universe of activation states. Progress has now been made in defining the signaling pathways, transcriptional networks, and epigenetic mechanisms underlying M1-M2 or M2-like polarized activation. Functional skewing of mononuclear phagocytes occurs *in vivo* under physiological conditions (e.g., ontogenesis and pregnancy) and in pathology (allergic and chronic inflammation, tissue repair, infection, and cancer) [54].

Macrophage subpopulations show different types of receptor expression and cytokine/chemokine production [55–59]. 

Classically activated macrophages, also called M1 cells, are induced by IFN-*γ* and have a high capacity to present antigen. They produce inflammatory cytokines such as IL-1*β*, IL-6, IL-12, IL-23, and TNF-*α* as well as high levels of inducible nitric oxide synthase (iNOS).

In contrast, alternatively activated macrophages, also called M2 cells, are induced by IL-4, which promotes type 2 responses [55].

M2 macrophages are characterized by efficient phagocytic activity, high expression of several receptors such as class A scavenger receptor (CD204), MR, dectin-1, CD209, CD163, production of ornithine and polyamines through the arginase pathway, and an IL-12^lo^IL-10^hi^IL-1decoyR^hi^IL-1RA^hi^ phenotype [54, 56, 60].

Chemokine receptors and ligands are differentially modulated in polarized macrophages. In particular, production of IFN-*γ*-induced protein of 10 kDa (IP-10/CXCL10) and monokine induced by gamma interferon (MIG/CXCL9) are inhibited by IL-4 and IL-10. However, IL-4 selectively induces eotaxin-2/CCL24, CCL18, and macrophage-derived chemokine (MDC/CCL22) in macrophages, and these effects are inhibited by IFN-*γ*. Therefore, differential production of chemokines that attract Th1 (CXCL9, CXCL10) and Th2 or T regulatory (Tr) cells (CCL22) integrates M1 and M2 macrophages in circuits of amplification and regulation of polarized T-cell responses [60].

M1 cells are related with chronic inflammation and tumor inhibition, while M2 cells are related with tumor cell growth and metastasis through angiogenesis and tissue remodeling [22]. Allergy is driven by Th2 cells and products and is associated with M2 polarization of macrophages [61–63]. IL-4-inducible chemokines acting on CCR4 (e.g., CCL22) have also been reported to promote skewing of macrophage function [64]. Evidence now indicates that chitin- and arginase-dependent M2 pathways play an active role in the pathogenesis of allergy [65]. Asthma is associated with tissue remodeling, including collagen deposition and goblet cell hyperplasia. IL-4-driven M2 polarization is likely to play a key role as an orchestrator of these processes [66]. Allergy represents a paradigm for IL-4/IL-13-driven type 2 inflammation. However, there is evidence that the inflammasome/IL-1/Th17 pathway can also drive allergic inflammation [67, 68]. Moreover, a Th1-associated cytokine, IL-18, has also been implicated in allergic inflammation [69]. 

Patients with AD exhibit exaggerated Th2 responses, and initiation of AD lesions is thought to be mediated by means of early skin infiltration of Th2 lymphocytes releasing high levels of IL-4, IL-5, IL-13, and IL-31 [53, 70, 71]. Subsequently, the accumulation of activated monocytes, mature DCs, and eosinophils determines a rise in IL-12 expression and the appearance of a mixed Th2/Th1 cytokine pattern, with reduced IL-4 and IL-13 and the presence of IFN-*γ* in the chronic phase [70, 71]. It is therefore perhaps not surprising that mixed phenotype macrophages (M2/M1) should be observed in AD which shows a mixed Th2/Th1 phenotype. However, there is no clear investigation in this area, and the exact mechanism of macrophages activation remains elusive. Further investigation should be performed to clarify the role of M2 and M1 in different phases of inflammation in AD.

In addition to the possible role of M2 and M1 macrophages in acute and chronic inflammation of AD, there are several molecules and factors (e.g., histamine, staphylococcal components, cAMP, and Fc*ε*RI ligation) which regulate cytokines and chemokines secretion through monocytes and macrophages in AD [72–75].

### 5.1. Histamine

For instance, histamine influences the profile of proinflammatory and immunoregulatory cytokines produced by blood monocytes, tissue macrophages, and DCs [76, 77]. Histamine induces the production of IL-10 and inhibits that of TNF-*α* and IL-12 from monocytes. Interestingly, histamine induces TNF-*α* production from macrophages [78] but not from monocytes or DCs [79, 80]. Although these observations indicate that histamine exerts important immunoregulatory effects, they also illustrate the heterogeneity of the responses of human immune cells to this mediator [79].

The biologic effects of histamine are mediated by activation of 4 distinct receptors (H1, H2, H3, and H4) [72]. In the context of AD, we recently showed that human monocytes express the H4R, and that its stimulation leads to a Ca^2+^ influx and an inhibition of CCL2 production, resulting in a reduction of monocyte recruitment [81].

This could represent a negative feedback mechanism to avoid an overwhelming Th2 environment in case of a persistent histamine release at sites of allergic inflammation and could contribute to the shift from Th2 to Th1 observed in the transition from acute to chronic allergic inflammation such as AD [81].

Furthermore, we showed that histamine downregulates IL-27 production in APCs including monocytes. The downregulation of IL-27 by histamine might be a new mechanism in the pathogenesis of inflammatory skin diseases, in particular, if increased concentrations of histamine are present at sites of inflammation, such as in chronic eczema [82].

### 5.2. Staphylococcal Exotoxins

Recent findings suggest a role for staphylococcal superantigens in the production of chemokines and cytokines during the development of atopic skin inflammation. 

First, superantigen exposure may directly lead to the production of chemokines by T cells, macrophages, and DCs (CCL1 or CCL18). Second, superantigens may induce the release of effector cytokines such as IL-4, IFN-*γ*, or IL-31 which in turn may upregulate the expression of chemokines such as CCL1, CCL11, CCL17, CCL18, CCL26, CXCL9, or CXCL10. These chemokines are mainly associated with macrophages. Third, IL-31-induced pruritus may be accompanied by skin injury through scratching resulting in the production of primary proinflammatory cytokines such as IL-1*α* and TNF-*α* which in turn may amplify chemokine production (e.g., CCL20 or CCL27) [53, 73].

In addition, we found that staphylococcal exotoxins (SEB or *α*-toxin) significantly upregulates IL-31 receptor A (IL-31RA) expression on monocytes and macrophages. Moreover, IL-31 induces proinflammatory cytokines in monocytes and macrophages following staphylococcal exotoxins stimulation. Such data provide a new link between staphylococcal colonization and the worsening of inflammation via IL-31 in monocyte and macrophages [83].

### 5.3. Cyclic Adenosine Monophosphate (cAMP)

Some studies have shown that leukocytes from patients with AD have increased levels of cAMP-phosphodiesterase activity which results in reduced intracellular cAMP, creating a permissive effect on cell function. This increased activity accounts for subnormal cAMP responses and correlates with increased PGE2 production of monocytes, which inhibits Th1 responses and accentuates IL-4 secretion by Th2 cells [74, 84]. 

### 5.4. Fc Epsilon Receptor I (Fc*ε*RI) Ligation

Fc*ε*RI ligation on monocytes of atopic donors induces indoleamine dioxygenase, which is similar to IL-10 in that it is involved in the control of T-cell responses and the induction of tolerance in the immune system [75].

The expression of Fc*ε*RI and Fc*ε*RII on monocytes in the peripheral blood is increased in atopic subjects and is significantly higher in patients with extrinsic AD than in patients with intrinsic AD. Recent concepts support the hypothesis that Fc*ε*RI-bearing monocytes in the peripheral blood might be the source of subtypes of IgE-bearing DCs in epidermal lesions of patients with AD, which are recruited in the acute phase or during exacerbation of the disease into inflammatory skin by chemokines, cytokines, and other mediators. Fc*ε*RI on APCs seems to play a pivotal role in modulating the differentiation [15, 75]. 

Taken together, these studies provide new insights in contribution of monocytes and macrophages in the complex network of cytokines and chemokines in AD as well as role of these cells in the amplification cycle of atopic skin inflammation.

## 6. Angiogenesis and Lymphangiogenesis

Angiogenesis and morphological and functional alterations of microvessels are hallmark features of chronic inflammatory disorders, including AD [85]. 

Vascular endothelial growth factors (VEGFs) are key regulators of blood vessel growth. The VEGF family includes VEGF-A, -B, -C, -D, and placental growth factor. VEGF-A and -B are the most important preangiogenic factors, while VEGF-C and -D primarily regulate lymphangiogenesis. High levels of VEGF-A have been detected in skin tissue of AD patients and correlate with disease activity. The vascular changes in the skin of AD patients appear to be linked to the inflammatory process. Besides human mast cells, basophils, eosinophils, and lymphocytes, macrophages as one of the effector cells of skin inflammation, are major sources of a vast array of angiogenic and lymphangiogenic factors such as VEGFs, angiogenin, and IL-8. 

Activated macrophages induce neovascularization and contribute to angiogenesis and lymphangiogenesis in inflammatory diseases. Primary human macrophages express angiogenic (VEGF-A and -B) and lymphangiogenic factors (VEGF-C and -D) [86]. 

Secretory phospholipases A2 enzymes present in the sites of inflammation enhance the expression and release of VEGF-A and -C in human macrophages [85, 86]. 

Using a bacterial pathogen-induced model of acute skin inflammation, it has been demonstrated that the lymphangiogenic growth factors (VEGF-A, -C, and -D) secreted from macrophages in inflamed skin tissue seem to be critical not only in lymphatic vessel expansion, but also in antigen clearance and inflammation resolution through enhancement of lymphangiogenesis [87]. The latter observation is interesting because AD patients have increased colonization and superinfection with *Staphylococcus aureus* [88]. There is some evidence that angiogenesis is dysregulated in humans and experimental models of AD. 

For instance, Shi et al. [89] investigated the possible link of macrophages recruitment and lymphangiogenesis in Keratin14-IL-4 Transgenic (Tg) mouse model of AD. They demonstrated that the density of VEGF-C-expressing CD11b^+^ macrophages increases significantly only within the dermis of lesional skin [89]. 

Their study suggests that CD11b^+^ macrophages might contribute to neolymphangiogenesis in AD by producing VEGF-C [89].

The possible role of lymphangiogenesis in different phenotypes and phases of AD remains elusive. It is likely that better understanding of altered angiogenesis/lymphangiogenesis in different forms and stages of AD may prove beneficial in the treatment of this common inflammatory skin disorder. 

It is possible that specific inhibitors of various mediators (VEGFs) and receptors (VEGFRs, Tie-2, etc.) controlling angiogenesis/lymphangiogenesis may offer novel strategies for dealing with treatment of microvascular changes in inflammatory skin disorders [85]. The possible relevance of angiogenesis/lymphangiogenesis in the pathophysiology and therapy of AD makes the study of vascular remodeling in this disorder a priority for future research.

## 7. Conclusions and Perspective

Much research effort over the last years has concentrated on the identification of dysregulated genetic and immunologic pathways that could lead to the manifestation of AD. Within this dense network of skin immune cells, APCs including macrophages play an outstanding role and are therefore at the center of focus. Macrophages are an essential component of innate immunity and play a central role in inflammation and host defense. Because of their versatile roles in the pathophysiology of AD, their multifaceted character, and their capacities to both promote and prevent the manifestation of allergic skin inflammation, macrophages represent promising cellular targets for therapeutic approaches in the future.

## Figures and Tables

**Table 1:** Characteristics of monocytes/macrophages-derived cytokines.

Cytokine	Structure	Size molecular weight	Receptors	Cell targets	Major functions	Disease association
IL-1*α*, IL-1*β*	Heterodimer	17 kd	IL-1RI, IL-1RII	T cells, fibroblasts, epithelial and endothelial cells	Induction of Proinflammatory proteins, hematopoiesis, differentiation of Th17 cells	Wide range of autoimmune and inflammatory diseases: AD, RA, IBD, psoriasis

IL-3	Monomer	15 kd	IL-3R*α* + *β*c (CD131)	Erythroid progenitors, Granulocyte macrophages progenitors, CD34+ progenitor cells, basophils, eosinophils	Hematopoietic growth factor, activation of basophils and eosinophils	Role in allergic diseases for example, AD, different types of cancers, lymphocytic and acute myeloid leukemias

IL-6	Homodimer	19–26 kd	IL-6R, (sIL-6R) gp130	Hepatocytes, leukocytes, T cells, B cells, hemopoietic cells	Liver: synthesis of acute phase proteins; leukocytes: trafficking, activation; T cell: differentiation, activation, survival; B cell: differentiation, production of IgG, IgM, IgA hematopoiesis	Autoimmune disease, chronic inflammatory diseases for example, AD, B-cell malignancy, SLE, Castleman's disease, plasmacytoma/multiple myeloma

IL-7	Monomer	25 kd	IL-7R and sIL-7R	B, T, and NK cells	Proliferation of pre-B and pro-B cells (mice), megakaryocytes maturation, VDJ recombinations, naive T-cell survival, synthesis induction of inflammatory mediators in monocytes	Allergy/autoimmunity and psoriasis

IL-8 (CXCL8)	Homodimer	16 kd	CXCR1 and CXCR2	Neutrophils, NK cells, T cells, basophils, eosinophils, endothelial cells	Chemoattractant for neutrophils, NK cells, T cells, basophils, eosinophils; mobilization of hematopoieticstem cells; angiogenesis	Increased levels during inflammatory diseases (e.g., AD, RA, psoriasis, bacterial and viral infections)

IL-10	Homodimer	20.5 kd, predicted size of precursor protein; 18.6 kd, predicted size mature protein, monomer	IL-10R1/IL-10R2 complex	Macrophages, monocytes, T cells, B cells, NK cells, mast cells, DC and granulocytes	Immune suppression	Cancer, autoimmunity, allergy (e.g., AD)

IL-12 (p35/p40)	Heterodimer	IL-12a p35, 35 kd; IL12b p40, 40 kd	IL-12Rb1 and IL-12Rb2	T cells (Th1 cells), NK cells	Induce Th1-cell differentiation and cytotoxicity	Chronic inflammation (e.g., AD), impaired Th1 response with higher susceptibility to intracellular pathogens, use as anticancer agent

IL-15	Monomer	14-15 kd	IL-15R	T, NK, and NKT cells T-cell activation	Proliferation and activation of NK cells, differentiation of *γ*/Δ T cells, suppression of IL-2 induced AICD of T cells, homeostasis of CD8+ memory, NK and NKT cells, enhancement of Th2 differentiation and suppression of allergic rhinitis	Autoimmune and inflammatory diseases

IL-16	Homotetramer	56 kd	CD4	T cells, monocytes, macrophages, eosinophils	Chemotaxis, modulation of T-cell response	Increased during various inflammatory and infectious diseases including *atopic * *eczema*, allergic asthma, Crohn's disease, RA, hepatitis C infection, tuberculosis; inhibits HIV infection

IL-18	Heterodimer	22.3 kd	IL-18R	Variety of cells, T cells, NK cells, macrophages, epithelial cells, chondrocytes	Induction of IFN-*γ* in presence of IL-12, enhances NK cell cytotoxicity, promoting Th1 or Th2-cell responses depending cytokine milieu	Autoimmune diseases or inflammatory disorders, AD, RA, psoriasis, MS, type I diabetes

IL-19	Monomer	20.5 kd predicted size of precursor; 17 kd, predicted size of mature protein; 35–40 kd, found in transfected cells, glycosylated	IL20R1/IL-20R2	Keratinocytes	Unknown	Psoriasis

IL-20	Monomer	20 kd predicted size of precursor; 17.5 kd, predicted size of mature protein	IL-20R1/IL-20R2 and IL-22R1/IL-20R2	Keratinocytes, monocytes	Role in skin biology	Psoriasis, RA, atherosclerosis

IL-23 (p191p40)	Heterodimer	IL-12b p40, 40 kd; IL-23 p19, 19 kd	IL-12Rb1 and IL-23R	T cells (TH17 cells) and macrophages	Stimulate production of proinflammatory IL-17 and promote memory T-cell proliferation	Susceptibility to extracellular pathogens, exacerbate organ specific autoimmune inflammation, chronic inflammatory diseases (psoriasis, AD)

IL-24	Homodimer and monomer	23.8 kd, predicted size of unprocessed precursor; 18 kd, unglycosylated mature protein; 35 kd, observed size of secreted IL-24, glycosylated	IL20R1/IL-20R2 and IL-22R1/IL-20R2	Keratinocytes, cancer cells	Tumor suppression	Melanoma, psoriasis

IL-27 (p281EBI3)	Heterodimer	IL-27a p28, 28 kd; IL-27b EBI3, 25.4 kd	WSX-1 and gp130	T cells, NK cells	Induction of Tbet promoting Th1-cell differentiation, inhibition of Th17-cell response via STAT1	Immune pathology because of uncontrolled inflammatory response: for example, in psoriasis or in epidermal compartment of *patients with eczema *

IL-32	Unknown	14.9–26.6 kd	Unknown	Macrophages, DCs, T cells, PBMCs, monocytes	Induction of TNF-*α*, IL-8, and IL-6, apoptosis	AD, RA, IBD, autoimmune disease

IL-37	Unknown	17–24 kd	IL-18Ra?	Intracellular mechanism manner and DC	Suppression of proinflammatory cytokines and inhibition of DC activation	RA

TNF-*α*	Homotrimer	26 kd transmembrane and a 17 kd secreted form	TNF-R1 and TNF-R2	Both receptors are virtually on all cell types except for the red blood cells, but TNFR1 is more ubiquitous, and TNFR2 is often more abundant on endothelial cells and cells of hematopoietic lineage	Regulation of immune cells: induce fever, apoptotic cell death, (through IL-1 and L-6 production, inhibit tumorigenesis and viral replication, recruiting macrophage and neutrophils to a site of infection	Chronic inflammation (AD, psoriasis, RA, IBD, COPD), Alzheimer's disease, cancer

AD:  atopic dermatitis; AICD: activation-induced cell death; COPD: chronic obstructive pulmonary disease; IBD: inflammatory bowel disease; Ig:  immunoglobulin; kd: kilo Dalton; NK:  natural killer; RA: rheumatoid arthritis; SLE:  systemic lupus erythematosus; sIL-6R: soluble interleukin-6 receptor; Th: T helper.

## Abbreviations

AD: Atopic dermatitis
APC: Antigen presenting cells
AMP: Antimicrobial peptide
cAMP: Cyclic adenosine monophosphate
CCL: Chemokine (C-C motif) ligand
CXCL: Chemokine (C-X-C motif) ligand
CCR: Chemokine (C-C motif) receptor
CD: Cluster of differentiation
DC: Dendritic cells
Fc*ε*RI: Fc epsilon receptor I
IDEC: Inflammatory dendritic epidermal cell
IFN-*γ*: Interferon-gamma
IL: Interleukin
IP-10: IFN-*γ*-induced protein of 10 kd
LC: Langerhans cells
LPS: Lipopolysaccharide
LTA: Lipoteichoic acid
MDC: Macrophages derived chemokine
MIG: Monokine induced by gamma interferon
MR: Mannose receptors
NK: Natural killer cells
NLR: Intracellular nucleotide-binding oligomerization domain receptor
NOD: Nucleotide-binding oligomerization domain
PAMP: Pathogen-associated molecular patterns
PBMC: Peripheral blood mononuclear cells
PRR: Pattern-recognition receptor
*S*. *aureus*: *St* *ap* *hy* *lo* *co* *cc* *us* *aureus*
SEB: Staphylococcal enterotoxin B
SNP: Single-nucleotide polymorphism
Th: T helper cells
TLR: Toll-like receptor
TNF: Tumor necrosis factor
VEGF: Vascular endothelial growth factor.
